# Ecological Risk and Pollution Assessment of Heavy Metals in Farmland Soil Profile with Consideration of Atmosphere Deposition in Central China

**DOI:** 10.3390/toxics12010045

**Published:** 2024-01-08

**Authors:** Yang Zhao, Yuxin Hou, Fei Wang

**Affiliations:** 1 School of Physical Education, Shanxi University, Taiyuan 030006, China; benkangarro@126.com (Y.Z.); hyuxin0103@163.com (Y.H.); 2 Sports Science Institute, Shanxi University, Taiyuan 030006, China

**Keywords:** heavy metal, farmland soil, atmosphere deposition, ecological risk, pollution assessment

## Abstract

Heavy metals (HMs) in agricultural land have caused serious environmental problems, resulting in severe contamination of the food chain and posing potential health threats. This study aims to investigate the pollution levels and potential ecological risks of HMs in farmland soils in central China, taking into account atmospheric deposition. Several indices were used to assess the status of HMs and compare surface soil with deeper soil. Descriptive statistics, Pearson correlation, and UMAP clustering methods were utilized to identify the characteristics of HMs. Additionally, stepwise linear regression models were employed to quantify the contributions of different variables to the potential ecological risks of HMs. The results showed that the average content of Zn in surface soil (289.41 ± 87.72 mg/kg) was higher than in the deeper soil (207.62 ± 37.81 mg/kg), and similar differences were observed in the mean values of related *I_geo_* (1.622 ± 0.453 in surface soil and 1.183 ± 0.259 in deeper soil) and *PEI* (0.965 ± 0.292 in surface soil and 0.692 ± 0.126 in deeper soil) indices. This indicates that surface soil is more heavily polluted. The UMAP results confirmed the high variability of HMs in the surface soil, while PCA results suggested the importance of pollution and ecological risk indices. The stepwise linear model revealed that different variable structures contribute differently to the risk. In conclusion, Cr and Zn were found to be the major contaminants in the local farmland soil, with higher concentrations in the surface soil. The geoaccumulation and total potential ecological risk were classified as low risk. High variability of HMs was observed in the surface soil. Therefore, HM-related pollution indices and ecological risk indices are important for assessing the contamination status of local HMs. The local potential ecological risk can be attributed to specific heavy metals, each of which can have different effects on the local ecological risk.

## 1. Introduction

Contamination of the environment with heavy metals (HMs) is a global issue that has gained significant attention over the past decade [1]. Soil pollution with toxic HMs has become more prevalent due to increasing human activity, urbanization, and industrialization [2]. HMs have been detected as widely distributed environmental pollutants due to extensive usage in industrial and daily consumer products [3,4,5,6,7]. HMs are reported in the literature as mostly toxic, persistent, and bioaccumulative [4,5,8,9]. They tend to accumulate as substances of variable risk in farmland soils [3] and transmit to the top parts of the food chain, which serve as bioaccumulators [10,11,12]. They accumulate in agricultural soils and can be transmitted through the food chain, resulting in contaminated food with harmful effects [13,14]. Furthermore, certain HMs such as Pb, Mn, Ni, Cd, and Cr have been listed as hazardous air pollutants by USEPA [15], which could aggravate soil pollution in farmland through atmospheric deposition.

The pollution of HMs in farmland soil is considered irreversible due to the stability and resilience of local geology, geographical characteristics, and local climate dynamics [16,17]. Moreover, HMs could be trapped when they interact with soil particles, forming chemical forms and/or metal speciation, which prolongs their presence in the soil and increases the risk of contamination [11]. Over time, atmospheric deposition of dust can contribute to the accumulation of HMs in soil, which is evident in the surface soil profile. Deeper soil layers, less influenced by atmospheric deposition, reflect naturally occurring elements from the local crust of the Earth. Additionally, human activities can disturb the soil and significantly increase the pollution level and spatial distribution, adding complexity to the overall system. 

Previous research has investigated HMs in agriculture and farmland soils [18,19,20,21,22]. These studies focused on various aspects, such as assessing the status of HMs [21,22], measuring contamination levels [3,19], and evaluating the ecological risk [2,16] or health impacts [3,15]. These studies utilized pollution or ecological risk indices combined with GIS techniques to create digital maps that allowed for visualizing the distribution patterns and relationships of HMs. Additionally, they examined larger areas, often with unique economic or geographical characteristics [23,24,25]. The initial assessment of HM contamination heavily relied on the spatial distribution presented in the GIS maps. The status and impacts of HMs on different depths of soil profiles should be given more attention. Mitran et al. revealed combined surface soil (0–15 cm) and depth-wise soil (15–30 cm, 30–50 cm, and 50–100 cm) monitoring of pollutant accumulation would be a valuable addition to choosing a reliable and practical approach to evaluate and gain a clear understanding of soil contamination [26]. It was crucial for decision making and reclamation planning of soil tillage. Huang et al. analyzed the chemical forms of heavy metals in different aggregate-sized fractions along the profile (0–1, 1–5, 5–15, and 15–25 cm) of a contaminated paddy field and revealed the exogenous metals of surface soil were first retained and then migrate to a deep layer through a leaching process, leading to a gradual decrease in metal concentration with soil depth [27]. One study by Luo et al. emphasized that urban surface soils are usually more contaminated owing to current human sources and these accumulated influences can also make deep soil layers contaminated by HMs [28]. The study by Kim et al. emphasized that the distribution patterns of soil constituents, especially HMs, within a soil layer should be carefully evaluated to help understand the soil contamination processes [29]. Currently, research on pollution has primarily focused on using a single factor index or a simple composite index. This has become the traditional approach in research. The existing methods for analyzing sources of HM pollution mainly involve geostatistical analysis and multivariate statistical analysis. However, the routine linear regression method only considers the performance of the model, without taking into account the construction of the model or the comprehensive impact of multiple variables. In the study, we use linear models and their comparisons after adjusting for different potential confounding factors to reveal the main integrated effects of HMs rather than the single effect. Thus, the objectives of the research include: (1) to explore the HM contamination status and levels; (2) to identify the influencing contributions of HM-related indices to the current HM status; (3) to quantify the effects of HM-related indices on local farmland ecological risk using linear models. 

## 2. Materials and Methods

### 2.1. Study Area

The study area is located in central China, which is the well-known main grain-producing area. The location is relatively flat, and the altitude range is less than 15 m. The average annual precipitation is less than 700 mm, and the average annual temperature is about 15 °C. The location belongs to the continental monsoon climate of the warm temperate zone and always has southerly wind in summer and a northerly wind in winter. The soil quality is formed by the alluvial of the Yellow River. The study area, due to less anthropogenic pollution impacting soil environment quality, is an important window for observing atmospheric deposition, as well as atmospheric particulate matter settlement monitoring. In a relatively smaller area with less than a 5 km radius, the monsoons and local climate change produce a homogenous impact. Against this consistent background, the content of heavy metals in the surface soil (0–10 cm) could represent the mixed impact of atmospheric deposition and soil, and the deeper soil (10–20 cm) could represent the local soil characteristic dynamics. 

### 2.2. Sample Collection and Analysis

**Figure 1:**
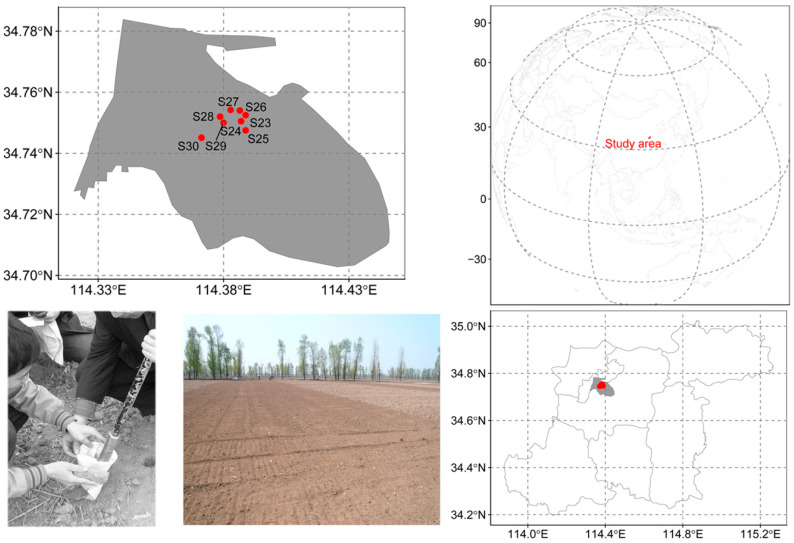
Geographical sites and in situ sampling of the study (gray area represents one city district).

The eight sampling sites (longitudes from 114.371° E to 114.404° E and the latitudes from 34.742° N to 34.766° N) of the study are used to explore the ecological risk and pollution assessment (Figure 1). The sampling data were collected in February 2019. Soil samples were randomly collected from 0 to 10 cm in the surface layer and 10–20 cm in the middle layer of landsoils. The surface layers of croplands from 0 to 10 cm were used to represent the mixed effects of atmospheric particulate matter settlement (APSM) on soil heavy metal dynamics. Accordingly, layers of landsoil profiles from 10 to 20 cm were used to represent the local soil effects (SOIL) of HM dynamics. 

The farmland heavy metal pollution risk items included Cd, As, Pb, Cr, Cu, Ni, and Zn. Before the lab detection processes, soil samples were air-dried at 25 °C, ground, sieved through 2 mm mesh in the laboratory, and stored in plastic bags. Then, 200 mg soil samples were digested in a dry and clean Teflon digestion beaker, and 8 mL HNO_3_, 5 mL HClO_4_, and 2 mL HF were added, and the mixture was heated for 40 min on a hot plate at 120–150 °C. The mixture was then filtered through Whatman filter paper and the filtered digest was transferred to a 50 mL plastic volumetric flask which was filled up to the mark by deionized water. Metal contents were measured by Agilent 700 series ICP-OES. A certified reference material (CRM) was used to validate the analytical measurement methods. All samples were replicated 3 times, and the average value was taken.

### 2.3. Methods of Ecological Risk and Pollution Assessment

The single factor pollution index method and Nemero comprehensive pollution index method could be used to assess the local farmland soil pollution levels [30]. The single factor pollution load index (*PI*) and integrated pollution load index (*PLI*) were employed to assess the pollution level of HMs in the soil samples of the studied area. The single factor pollution load index was determined using Equation (1).

*PI* = *C_i_*/*S_i_* (1)

where *PI* is the single factor pollution load index for the examined HMs; *C_i_* is the concentration of HMs in a soil sample (mg kg^−1^); and *S_i_* is the permitted standard of the same metal (mg kg^−1^) [24]. The average pollution index (*PI*) was calculated by the average of 7 heavy metal *P_i_,* which represents the equal weighted pollution of each heavy metal. The grading criteria were set to 1, 2, 3, and the pollution evaluation corresponded to four levels from non-pollution, light pollution, and medium pollution to heavy pollution [31].

The *PLI* represented by the Nemero comprehensive pollution index method was calculated as follows:

(2) $$PLI=\sqrt{\frac{1}{2}\left(\;{\left[\frac{1}{n}\sum_{i=1}^{n}PI\right]}^{2}+[P_{imax}^{2}]\right)}$$

where the *P_imax_* is the maximum value of *P_i_*. If the *P_i_* value is greater than unity, it suggests the existence of pollution or the presence of pollutants, while no pollution loads are inferred with a lower value. Among them, the grading criteria were set to 0.7, 1, 2, 3, and the pollution evaluation corresponded to five levels from safety, alert, light pollution, and medium pollution to heavy pollution [31]. Both the single factor pollution index and the integrated pollution load index could provide a more comprehensive reflection of the soil environmental quality in the study area.

Heavy metals in soils were also evaluated using the geoaccumulation index (*I_geo_*) [32]. It is expressed as:

*I_geo_* = *log*_2_ [*C_n_*/(1.5 *B_n_*)] (3)

where *C_n_* is the measured concentration of the examined metal *n* in the soil, and *B_n_* is the geochemical background concentration (or reference value) of the metal *n*. The constant 1.5 accounts for the natural fluctuations of the metal in soil. The geochemical background concentration (or reference value) of each metal was provided by local environmental government and the relevant statistical government. The background concentrations of Cr, Ni, Cu, Zn, As, Cd, Pb were set to 190, 100, 300, 25, 0.6, 170 mg/kg, respectively. The mean contents of the global geochemical background of tide soil and the average crustal abundance were used. The *I_geo_* has seven grades (0 to 6), indicating various degrees of enrichment above the background values and ranging from unpolluted to heavily polluted soil quality [33].

The total potential ecological risk index (*TEI*) is based on the necessary test item—farmland pollution risk screening value in the trial implementation of standards for pollution risk control of farmland for soil environmental quality. Hakanson’s potential ecological risk index method evaluated the ecological risk of heavy metal pollution in farmland soil in this area. The classification evaluation standard of the single potential ecological risk (*PEI*) of each heavy metal was established [34]. The calculation formulas of *TEI* and *PEI* are as follows:

(4) $$TEI=\sum_{n=1}^{i}\left(P_{i}\times T_{r}^{i}\right)$$

(5) $$PEI=P_{i}\times T_{r}^{i}$$

where is the corresponding toxicity coefficient of heavy metal element i (*T^i^*, constant for Cd is 30, As is 10, Pb is 5, Cr is 2, Cu is 5, Ni is 5, and Zn is 1). The grading ecological risk criterion of *TEI* was set to 150, 300, 600, and the pollution evaluation corresponded to four levels from low risk, moderate risk, and high risk to very high risk [31]. The grading ecological risk criterion of *PEI* was set to 40, 80, 160, and the pollution evaluation corresponded to four levels from low risk, moderate risk, and high risk to very high risk [31]. 

### 2.4. Statistical Method

Continuous numeric variables including HM concentration, *I_geo_* indices, *PEI* matrix, and pollution indices were expressed as the mean ± standard deviation for APMS and SOIL, respectively. Correspondingly, the quantile statistics were also calculated. The *t*-test was used to identify the statistical significance of the mean value variance of HMs between APMS and SOIL. The UMAP method was used in supervised clustering analysis, and the PCA method was explored to identify the important influencing factors. The stepwise linear regression models (step-lm) were explored to identify the effects of HMs with different adjustments for *I_geo_*-related variables, *PEI*-related variables, and pollution indices, respectively. The corresponding generalized linear mixed models (GLMMs) used random items of HM types (APMS and SOIL). However, the GLMM results were insignificant with tep-lm. Moreover, the variance decomposition could identify the effect of each variable. All statistical analyses and diagrams were conducted using R (version R 4.2.0, https://cran.r-project.org), with data cleaning and collation performed using the “tidyverse” package, descriptive statistics using the “gtsummary” package, and correlation calculation and drawing based on “ggcor” and “ggplot2” packages. The supervised clustering analysis and PCA were conducted with “tidymodels” packages. Standardization was required before data analysis, as well as the dummy variable settings. The stepwise linear model based on the standardized data was conducted by the “stats” package of R. All plots were drawn based on the “ggplot2” package of R.

## 3. Results and Discussion

### 3.1. Descriptive Statistics

The summary of the descriptive statistical analysis of heavy metal concentrations in APMS and SOIL is presented in Table 1. The mean concentrations of HMs in APMS are higher compared to SOIL, as well as in the *I_geo_*-related variables and *PEI*-related variables. The pollution indices also indicate higher levels of pollution in APMS. Specifically, the mean concentration of Zn in APMS is significantly higher (289.41 ± 87.72 mg/kg) compared to SOIL (207.62 ± 37.81 mg/kg) with a *p*-value of less than 0.05. A significant difference was also observed in *I_geo__Zn* and *PEI*_Zn. The *PLI* exhibited a significant difference between APMS and SOIL (*p* < 0.05). Moreover, only Cu and its associated *I_geo_* and *PEI* variables demonstrated lower values in the comparison between median and mean of APMS and SOIL. These findings indicate a greater disparity in quantiles between APMS and SOIL, with much higher values in APMS. The results suggest clear concentration effects of HMs due to the settling of atmospheric particulate matter, particularly in the case of Zn. The mean value of *PI* was 1.448 ± 0.343 in APMS and 1.274 ± 0.340 in SOIL, indicating light pollution in the study area. Meanwhile, the mean value of *PLI* was 3.010 ± 0.463 in APMS and 2.521± 0.232 in SOIL, indicating heavy pollution in APMS and medium pollution in SOIL, respectively. The *TEI* values were all less than 150, indicating a low ecological risk in the study area. However, *PEI* values grater than 40 were only observed for Cd, indicating a moderate risk (other *PEI*-related HMs exhibited a low potential ecological risk). The report by Wang et al. [35] indicates the moderate pollution of Cd with 41 samples, which has a similar result to our research. The research also found the Cd concentration in the atmosphere around the area is generally high, which enters the soil through rainfall or sedimentation, resulting in the higher Cd concentration in surface soil. Similarly, there is a potential ecological risk of Cd in both surface soil and deeper soil. The results by Al-Taani indicated a higher geoaccumulation of Cd and Ni than other HMs with 31 samples [2]. This result is also proved by our results. In addition, the relatively higher concentrations of HMs in surface farmland soil in our study are supported by Duan’s research [36]. It should be highlighted that higher geoaccumulation of Cd and Ni just can show the accumulation degree by comparing the contents obtained with geochemical baseline concentrations [37]. The excessive use of fertilizers and pesticides directly leads to the accumulation of Ni and Cd in the soil, and the high content in organic fertilizers also affects the degree of accumulation in farmland soil [38].

### 3.2. Correlation Analysis

**Figure 2:**
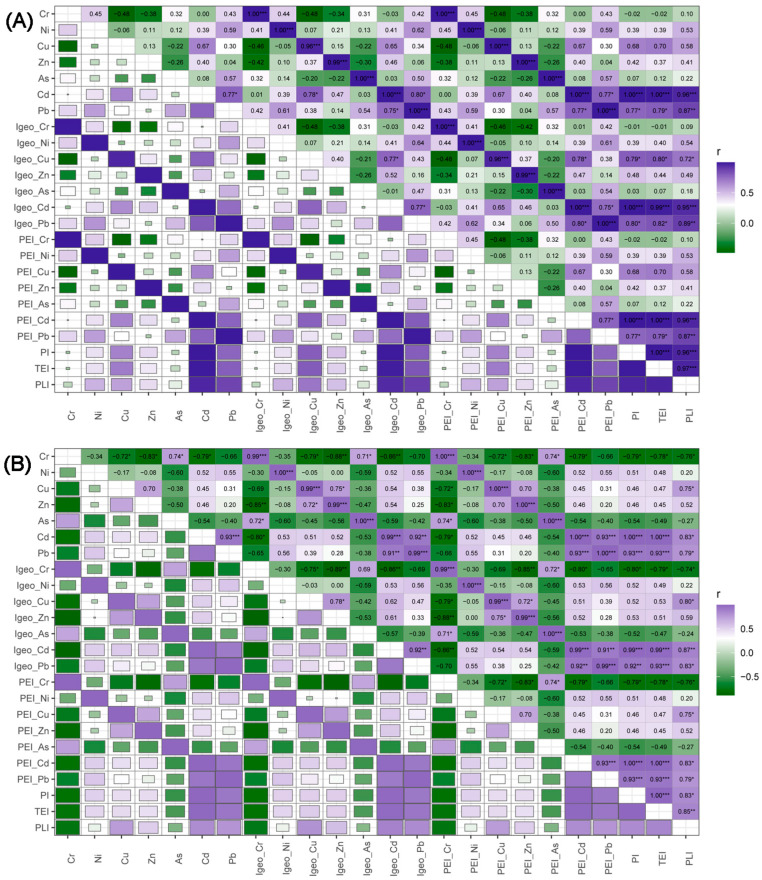
Correlation heatmap for APMS (**A**) and SOIL (**B**). (Significant statistialstatistical levels: * *p* < 0.05; ** *p* < 0.01; *** *p* < 0.001.)

All numeric variables were used to conduct the Pearson correlation analysis. The correlation heatmap displayed these relations among HMs, the *I_geo_*-related matrix, the *PEI*-related matrix, and pollution indices in APMS and SOIL (Figure 2). Clear differences were observed between APMS and SOIL. The correlation coefficient was higher in APMS compared to SOIL. In SOIL, there were more significant correlations between HMs, as well as among *I_geo_*-related and *PEI*-related variables. Significant correlations were also found among three pollution indices in both APMS and SOIL (*p* < 0.05). In SOIL, Cr showed significant correlations with other HMs, except Ni and Pb (*p* < 0.05), and Pb exhibited an extremely significant correlation with Cd (*p* < 0.001, Figure 2B). However, similar changes were not observed in APMS. Additionally, Cd, Pb, and their corresponding *I_geo_* and *PEI* values showed significant correlations with pollution indices. The previous research by Wang et al. [35] indicates that there is an obvious difference in HM concentration and spatial distribution between surface soil and deeper soil in the farmland of central China. Shao et al. indicated that atmospheric deposition originating from intensive coal combustion is considered the main source of HMs in the topsoil [39] which implies higher variations of HMs in surface soil. These differences could be reflected by correlation results to a certain extent, especially in deeper HM-contaminated soil.

### 3.3. Supervised Clustering Analysis

**Figure 3:**
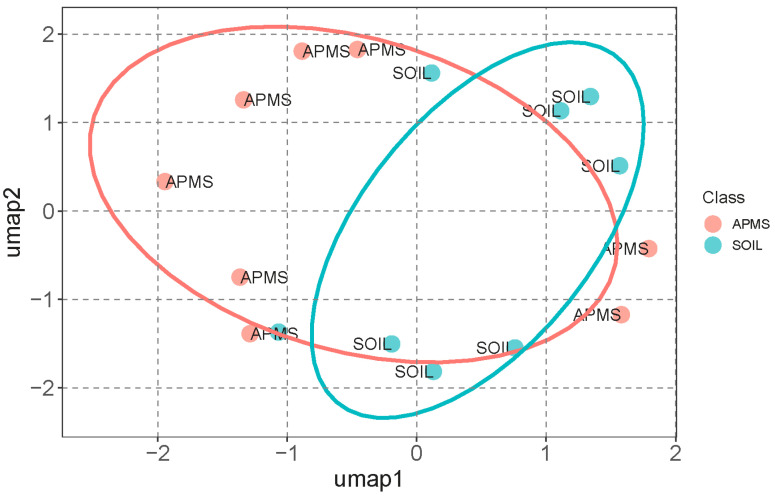
Clustering results of HMs and their related variables in APMS and SOIL.

The UMAP clustering results for HMs and their *I_geo_*-related variables, *PEI*-related variables, and pollution indices in APMS and SOIL revealed that HMs and their related variables can be grouped together. Among these, HMs in SOIL demonstrated the strongest clustering effect, with a relatively smaller 90% confidence ellipse (Figure 3). The results indicate that external interference is weaker in SOIL compared to APMS. This can be attributed to the higher influence of atmosphere decomposition and human activities on the surface soil. Furthermore, some individual sites showed mixed effects, likely due to their proximity to both SOIL and APMS sites. Similarly, two APMS sites displayed a significantly larger distance compared to the other APMS sites. The results from the in-depth distribution of HMs in southern Kazakhstan indicate the higher spatial distribution of HMs in surface soil [40]. Our clustering results also implied the relatively wider spatial distribution of HMs in farmland soil, especially in surface soil. Research assessed the effect of HMs in dust on landsoil, and the results indicate higher variability of HMs in dust [41]. Our results also support these high variabilities of HMs in atmospheric particulate matter settlement.

### 3.4. PCA

**Figure 4:**
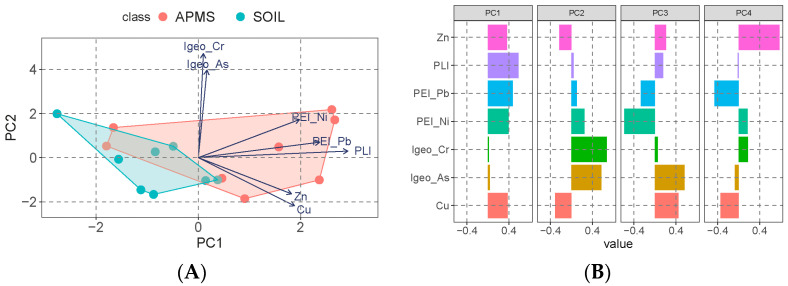
PCA result (**A**) and the most influencing variables in the first four axes (**B**).

The PCA results of the data from HMs and their *I_geo_*-related variables, *PEI*-related variables, and pollution indices indicate that Cu, Zn, *I_geo__Cr*, *I_geo__As*, *PEI_Ni*, *PEI_Pb*, and *PLI* are the most important influencing variables, as indicated by the relatively longer arrows (Figure 4A). Figure 4B provides a detailed breakdown of the most influencing variables in the first four axes. Within the components of the first axis, *PLI* has the strongest effect, followed by *PEI_Pb* and *PEI_Ni*, with Cu and Zn following. In the components of the second axis, *I_geo__Cr* and *I_geo__As* have the dominant effects. Furthermore, the similar clustering characteristics of HMs in SOIL indicate that it has the best clustering effect with a relatively smaller confidence boundary (Figure 4A).

The PCA results showed that the variables related to pollution or ecological risk had the greatest contribution in the first principal component (PC1). This was represented by *PLI*, *PEI_Pb*, and *PEI_Ni*. In the second principal component (PC2), the variables related to *I_geo_* were represented by *I_geo__Cr* and *I_geo__As*. These results implied the importance of pollution or ecological risk other than the HMs themselves. Liu et al. [42] reported ecological risk assessment and pollution classification should be given in more concentrations than each heavy metal content. As it should be, the solo heavy metal contamination in our study indicates the special spotlight of Cu and Zn. Other research using PCA indicates different HMs in different areas [19,42,43]. 

### 3.5. Stepwise Linear Multivariate Regression Model

The stepwise linear model with data standardization was explored to distinguish the individual effect from HMs (Table 2). By adjusting for potential confounding factors, the model’s performance improved. Four models were estimated for the stepwise linear regression analyses. Model 0 represented the linear response of the dependent variable *TEI* to the independent variables of HMs. Model 0 included components of HMs (not all, due to collinearity elimination in the step-lm model) in both APMS and SOIL that predicted the total ecological risk. Model 1 added *I_geo_*-related variables to Model 0, and Model 2 added *PEI*-related variables to Model 0. Finally, Model 3 investigated other pollution indices based on Model 0. Table 2 displays the adjusting variables, with N indicating “NO” and Y indicating “YES”. Furthermore, a generalized linear mixed model was conducted to identify any random effects due to the difference between APMS and SOIL. However, the model results did not show any significant effect from these random items. The model comparison also revealed no significant difference from the step-lm model, although it still allowed for variable decomposition to identify the importance of variable contribution.

The results of Model 0 indicate significant effects of Ni (*p* < 0.05) and Cu (*p* < 0.01) on local ecological risk. Zn, on the other hand, has a non-significant contribution (*p* > 0.05), and the model performance explained 52.09% of the variance. After adjusting for *I_geo_*-related variables in Model 1, significant contributions were found for Cu (*p* < 0.01) and Zn (*p* < 0.05), as well as significant contributions from *I_geo__Pb* and *I_geo__Cu*. The model performance improved, explaining 89.88% of the variance, compared to Model 0. After further adjusting *PEI*-related variables, based on Model 0, the results only showed an extremely significant contribution of Pb (*p* < 0.01) to local ecological risk. The contributions of *PEI_Cu* and *PEI_Zn* followed. The model performance achieved a notable improvement, with an adjusted R^2^ of 92.01% compared to 52.09% in Model 0. Finally, after adjusting pollution indices, the results showed extremely significant contributions of Cu, Zn, As, and Ni (*p* < 0.001) to local ecological risk, with the largest contribution from *PI*. The model performance had an adjusted R2 of 99.99%.

The comparisons between models can reveal the influence of adjusting variables on the single variable effect. In terms of the local ecological effects, the effect related to *I_geo_* show a relatively decreasing effect of Cu but an increasing significant effect of Zn. Additionally, the contributions of *I_geo__Pb* and *I_geo__Cu* are greater. The effect of *PEI* on local ecological risk indicates that the effects of Cu and Ni have disappeared, while the effects of Pb are extremely significant. Furthermore, *PEI_Cu* and *PEI_Zn* also have a huge effect. The pollution effects on local ecological risk reveal a decrease in the effects of Cu and Ni, but the addition of a negative significant effect of Zn and a positive significant effect of As. Additionally, the contribution of *PI* is extremely significant. These results show the associated influencing effects of *I_geo_*-related variables and *PEI*-related variables with HMs on local ecological risk, as well as the pollution effect of HMs. 

Although each site may pose a relatively small potential ecological risk, the different variable structures have varying impacts on the total potential ecological risks. The local total potential ecological risk is influenced by different variables related to HMs. Our research at least demonstrates that the local potential ecological risk of HMs can be explained partially by certain stubborn heavy metals, but the pollution associated with each heavy metal can also have different effects on the local ecological risk. Current research primarily focuses on HMs themselves [2,3,11,44], with less consideration for the simultaneous effects of their multiple related variables. However, HMs and their composite indices provide different insights into the effects of HMs. Each in situ heavy metal content or derived single variable only offers limited information on pollution or the relative risk of HMs [23]. Sun et al. [45] explored the health risks posed by heavy metals and other pollutants at various pollution levels using a multilinear model. Their results provide a deeper understanding of the effects of HMs on health and risk. Our results further establish a connection between HM pollution and their potential ecological risk, providing a way or pattern to gain a broader understanding of the effects and influences of HMs.

## 4. Conclusions

This study provided valuable information of farmland soil heavy metal contaminants with consideration of atmosphere deposition. Several indices of *PI*, *PLI*, *I_geo_*, *PEI*, and *TEI* were used to assess the HM status and the effects. Moreover, the stepwise linear model was used to quantify and discriminate the effects of multiple HM-related variables on *TEI*. Cr and Zn were the major contaminants in the local farmland soil, with high values of 54.62 ± 12.72 and 289.41 ± 87.72 mg/kg in surface soil. The geoaccumulation and total potential ecological risk were mostly at low risk levels (with *I_geo_* less than 3 and *TEI* less than 150). High variability of HMs is shown in surface soil. The HM-related pollution or ecological risk may have more importance. The local potential ecological risk of HMs could be explained by stubborn heavy metals, but the related pollution derived from each heavy metal could also bring different effects on local ecological risk. Restricted by region and sample size, more samples with long-term detection in future research are necessary which could further improve the accuracy and reliability of research for revealing the fate of HMs with more convincing proof. More samples covering more areas should be included in future prospective research. The study provides us with a way or pattern to further understand the effects or influences of HMs with wider thinking, and it will also help to improve the analysis accuracy further not only for researchers but also for policymakers.

## Figures and Tables

**Table 1:** Descriptive statistics of study variables.

Types	Variables	APMS (*n* = 8)				SOIL (*n* = 8)				*p*-Value
		Q1	Median	Q3	Mean (SD)	Q1	Median	Q3	Mean (SD)	
HMs (mg/kg)	Cr	43.475	59.197	65.058	54.62 (12.72)	36.215	43.899	48.2	45.03 (11.27)	*0.248*
	Ni	188.807	205.486	213.541	205.84 (22.42)	186.68	191.45	200.75	193.73 (10.51)	*0.208*
	Cu	48.491	57.165	65.657	66.45 (40.14)	36.916	49.135	55.51	45.45 (13.93)	*0.248*
	Zn	215.398	293.34	350.096	289.41 (87.72)	185.93	211.54	215.75	207.62 (37.81)	*0.046*
	As	14.47	15.147	15.882	15.732 (2.634)	11.965	13.015	16.322	14.33 (3.26)	*0.248*
	Cd	0.892	1.243	1.323	1.129 (0.282)	0.851	0.949	1.102	0.999 (0.291)	*0.529*
	Pb	27.431	32.202	39.958	34.397 (8.949)	25.93	28.68	35.981	31.04 (8.421)	*0.401*
*I_geo_*	*I_geo_* *_Cr*	−1.123	−0.68	−0.54	−0.830 (0.361)	−1.386	−1.109	−0.974	−1.107 (0.335)	*0.248*
	*I_geo_* *_Ni*	2.066	2.188	2.244	2.183 (0.152)	2.05	2.086	2.154	2.101 (0.078)	*0.208*
	*I_geo_* *_Cu*	0.68	0.952	1.129	0.982 (0.757)	0.317	0.724	0.91	0.549 (0.515)	*0.248*
	*I_geo_* *_Zn*	1.252	1.702	1.957	1.622 (0.453)	1.044	1.23	1.259	1.183 (0.259)	*0.046*
	*I_geo_* *_As*	−0.242	−0.175	−0.108	−0.136 (0.222)	−0.516	−0.394	−0.07	−0.286 (0.312)	*0.248*
	*I_geo_* *_Cd*	3.005	3.485	3.574	3.301 (0.398)	2.937	3.093	3.296	3.116 (0.425)	*0.529*
	*I_geo_* *_Pb*	−0.1	0.117	0.443	0.186 (0.363)	−0.182	−0.04	0.283	0.033 (0.385)	*0.401*
*PEI*	*PEI_Cr*	0.348	0.474	0.52	0.437 (0.102)	0.29	0.351	0.386	0.360 (0.090)	*0.248*
	*PEI_Ni*	4.969	5.408	5.62	5.417 (0.590)	4.913	5.038	5.283	5.098 (0.277)	*0.208*
	*PEI_Cu*	2.425	2.858	3.283	3.323 (2.007)	1.846	2.457	2.776	2.273 (0.697)	*0.248*
	*PEI_Zn*	0.718	0.978	1.167	0.965 (0.292)	0.62	0.705	0.719	0.692 (0.126)	*0.046*
	*PEI_As*	5.788	6.059	6.353	6.293 (1.054)	4.786	5.206	6.529	5.730 (1.304)	*0.248*
	*PEI_Cd*	44.576	62.158	66.128	56.45 (14.09)	42.53	47.434	55.093	49.94 (14.56)	*0.529*
	*PEI_Pb*	0.807	0.947	1.175	1.012 (0.263)	0.763	0.844	1.058	0.913 (0.248)	*0.401*
Index	*PI*	1.168	1.588	1.681	1.448 (0.343)	1.098	1.218	1.399	1.274 (0.340)	*0.401*
	*TEI*	60.787	78.915	84.978	73.89 (16.12)	57.592	62.059	71.442	65.01 (14.56)	*0.345*
	*PLI*	2.67	3.131	3.383	3.010 (0.463)	2.429	2.543	2.666	2.521 (0.232)	*0.046*

**Table 2:** The stepwise linear model summary of *TEI* and other HM-related variables under different variable adjustment.

Variables	Model 0	Model 1	Model 2	Model 3
Cr	——	——	——	——
Ni	0.427 (0.179) *	——	——	0.024 (0.002) ***
Cu	0.610 (0.179) ***	0.3026 (0.092) **	——	0.067 (0.003) ***
Zn	——	0.206 (0.088) *	——	−0.013 (0.002) ***
As	——	——	——	0.066 (0.002) ***
Cd	——	——	——	——
Pb	——	——	0.7372 (0.078) ***	——
*PI*	——	——	——	0.963 (0.003) ***
*PLI*	——	——	——	——
**Model Performance**				
Adj.R^2^	52.09%	89.88%	92.01%	99.99%
AIC	53.59	41.718	13.051	−26.798
BIC	59.771	50.989	21.55	−19.844
Adjust *I_geo_*	N	Y	N	Y
Adjust *PEI*	N	N	Y	N
Adjust *PI* + *PLI*	N	N	N	Y
Variable Importance	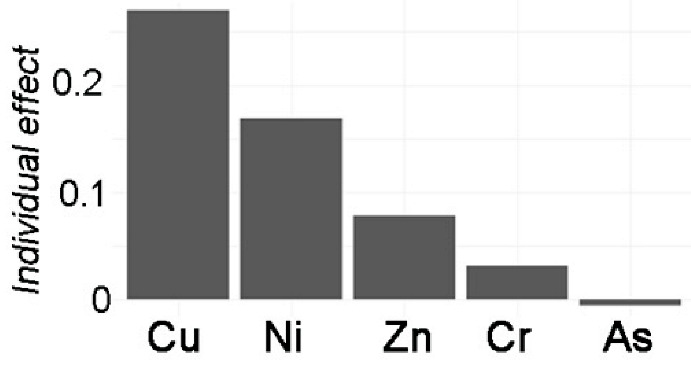	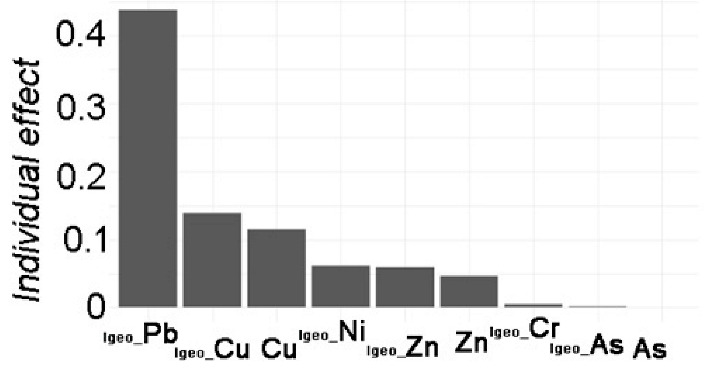	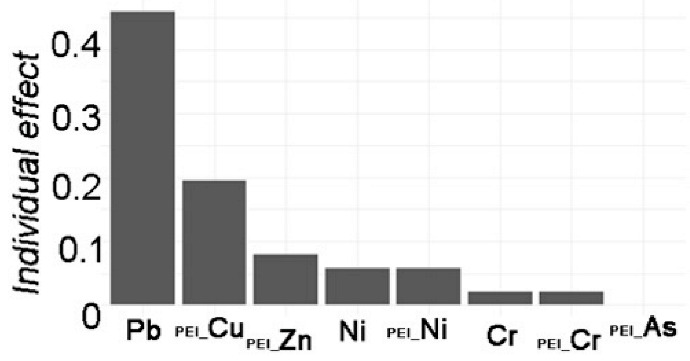	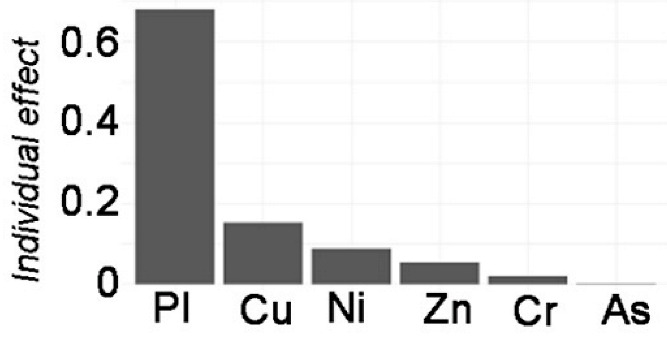

* Significant statistical levels: *p* < 0.1; * *p* < 0.05; ** *p* < 0.01; *** *p* < 0.001.

## Data Availability

The data and R codes that support the findings of this study are available on request from the corresponding author.
